# Genomic amplification of the human telomerase gene (hTERC) associated with human papillomavirus is related to the progression of uterine cervical dysplasia to invasive cancer

**DOI:** 10.1186/1746-1596-7-147

**Published:** 2012-10-30

**Authors:** Hongqian Liu, Shanling Liu, He Wang, Xiaoyan Xie, Xinlian Chen, Xuemei Zhang, Youcheng Zhang

**Affiliations:** 1 Department of Obstetric and Gynecologic, West China Second University Hospital, Sichuan University, Chengdu, 610041, China; 2 Laboratory of Cell and Gene Therapy, West China Institute of Women and Children's Health, West China Second University Hospital, Sichuan University, Chengdu, 610041, China; 3 Laboratory of Genetics, West China Institute of Women and Children's Health, West China Second University Hospital, Sichuan University, Chengdu, 610041, China; 4 Key Laboratory of Obstetric & Gynecologic and Pediatric Diseases and Birth Defects of Ministry of Education, Chengdu, 610041, China

**Keywords:** Cervical cancer, Telomerase, hTERT, hTERC, HR-HPV

## Abstract

**Background:**

Human papillomavirus (HPV) infection plays an etiological role in the development of cervical dysplasia and cancer. Amplification of human telomerase gene (hTERC) and over expression of telomerase were found to be associated with cervical tumorigenesis. This study was performed to analyze genomic amplification of hTERC gene, telomerase activity in association with HPV infection in different stages of cervical intraepithelial neoplasia (CIN) and cervical cancer. We were studying the role of hTERC in the progression of uterine cervical dysplasia to invasive cancer, and proposed an adjunct method for cervical cancer screening.

**Methods:**

Exfoliated cervical cells were collected from 114 patients with non neoplastic lesion (NNL, n=27), cervical intraepithelial neoplasia (CIN1, n=26, CIN2, n=16, CIN3, n=24) and cervical carcinoma (CA, n=21), and analyzed for amplification of hTERC with two-color fluorescence in situ hybridization (FISH) probe and HPV-DNA with Hybrid Capture 2.

From these patients, 53 were taken biopsy to analyze telomerase activity by telomeric repeat amplification protocol (TRAP) and expression of human telomerase reverse transcriptase (hTERT), with immunohistochemistry (IHC). All biopsies were clinically confirmed by phathologists.

**Results:**

Amplification of hTERC was significantly associated with the histologic diagnoses (p<0.05). The positive correlation was found between the level of hTERC amplification and histologic grading of dysplasia (CIN2/3 from CIN1 or normal, P=0.03). A profounding increase in the accumulation of HPV and hTERC positive cases was observed in the CIN3 subgroup compared with the CIN2 group, 25% versus 62.96%, respectively (p=0.007).

**Conclusions:**

hTERC ampliffication can be detected with FISH technique on exfoliated cervical cells. Amplification of hTERC and HPV infection are associated with more progressive CIN3 and CA. The testing of hTERC amplification might be a supplementary to cytology screening and HPV test, especially high-risk patients.

**Virtual slides:**

The virtual slide(s) for this article can be found here: http://www.diagnosticpathology.diagnomx.eu/vs/1857134686755648.

## Introduction

Cervical cancer (CC) is the second leading cause of cancer deaths in women, with more than 80% of these occurring in developing countries that have limited access to screening programs. It is estimated that 12,170 women will develop cervical cancer and about 4,220 women will die from cervical cancer in the United States during 2012 [1]. And in China, the mortality rate of cervical cancer ranged from 2 to 4 per 105 population in urban areas, 0 to 7 per 105 population in rural areas during the period of 1996 to 2005 [2]. Remarkably, it always takes about ten years to arise from precancerous lesion to invasive cervical cancer. For this reason, the effective screening of precursor lesion is of great importance, which makes cervical cancer preventable and curable. Cytological examination and HPV test are the most widely applied screening methods for CC and its precursor (cervical intraepithelial neoplasia, CIN) lesion. Although the implementation of the Papanicolaou test has been in routine, the test has limited accuracy not likely to be improved with enhanced collection and screening procedures. Specifically, the inability to distinguish high-grade CIN with potential possibility of progression to invasive cancer from pathologically insignificant or regressing dysplasia contributes to overtreatment, whereas false-negative results could not been eliminated. Furthermore, although 95% of patients with precancerous lesions harbour oncogenic HPV, only a small fraction of these eventually progresses to invasive carcinoma (invCA) [3]. Progression of a high-risk HPV infected CIN lesion to invCA is a result of further specific genetic alterations within the host cell genome. One of the consequences that are crucial for tumor development is an increased activity of telomerase.

Telomerase is a ribonucleoprotein enzyme complex that adds 5^′^-TTAGGG-3^′^ repeats onto the ends of human chromosomes, providing a telomere maintenance mechanism for about 90% of cancers [4]. Telomerase consists of several subunits, including hTERC that serves as a template during telomere elongation and a catalytic subunit, human telomerase reverse transcriptase (hTERT), which has reverse transcriptase activity. The amplifying of hTERC gene can stop cell apoptosis, consequently leading to tumor occurrence. Now we know that the detection of gain of hTERC may make clear distinguish between cytologically low-grade precancerous lesions and high-grade lesions, reaching sensitivity and specificity of over 90% [5,6].

Tumorigenesis is a complex phenomenon, whose manifestations may appear simultaneously on multiple levels, the genomic, proteomic, and functional alternation. The correlation between telomerase activation during cellular immortalization and in tumor progression suggests that telomerase activation is required for tumor growth. Moreover, telomerase activity is dependent on the expression of 2 main core component genes, hTERT and hTERC. This has prompted a large number of studied addressing telomerase activity using TRAP assay [7,8], amplification of hTERC using in situ hybridization [5,6,9-12] or expression of hTERT using IHC in CC and CIN lesions [13-15]. Cluster analysis between methods applied to CIN have shown that each level (phenotype, genotype, progression) should not be studied by itself, as there may be causal or linear relationships between them, which could explain more extensively and accurately the tumorigenesis pathway as a dynamic process [16]. Until now, however, there is no one study that has been designed to detect these three biomarkers in one research in clinical samples from CC and CIN lesions.

In this study, we observed, in a large sample scale, amplification of hTERC for progression from normal cervical tissues to dysplastic lesions and to invasive carcinomas in Chinese women. And we investigated gain of hTERC, expression of hTERT, and telomerase activity, and assessed associations among them. In addition, we assessed gain of hTERC in relation to physical status of HR-HPV infection. The results were analyzed to gain insights into the potential possibility for future clinical applications.

## Materials and methods

### Samples and cytological screening

Total of 114 outpatient or inpatient patients, visiting physician office at the Department of Obstetrics and Gynecology of West China Second University Hospital, Sichuan University from year 2007 to 2008, were included in this study with colposcopy-directed biopsy or hysterectomy. All participants voluntarily signed the consent form and completed a questionnaire. All patients had been swapped to get exfoliated cervical cells, which were collected by saline, for FISH test. Cell samples also had been taken for high-risk HPV HC2 test in the Prenatal Diagnosis Center, West China Second University Hospital, Sichuan University, China. Biopsy tissues from 53 patients were taken during entry colposcopy (EC) or surgery. Each tissue was divided into two parts, one was freezed in −70°C immediately, and another fixed in 10% neutral buffered formaldehyde solution for embedding in paraffin.

The histological diagnosis and grading of cervical cancers was established according to the guidance of International Federation of Gynecology and Obstetrics. Cervical cytology and histology were initially diagnosed by pathologists at the Center and then reviewed by a panel of expert pathologists. Histological diagnosis was made based on biopsy, endocervical curettage, and/or excision procedure. All 114 samples consisted of 27 normal or inflammatory specimens, 26 CIN1, 16 CIN2, 24 CIN3, and 21 invasive cervical carcinomas. The latter included 18 squamous cell carcinomas, 2 mucinous adenocarcinomas and 1 minimal deviation adenocarcinoma. HPV DNA testing was performed, blind to histologists, in the local Clinical Laboratory, using Hybrid Capture 2 (HC2, Digene Corporation, Gaithersburg, MD, USA). The normal group, as the control, was matched for age and ethnicity/race with study groups.

### Fluorescence *in situ* hybridization (FISH) for hTERC

The specific hTERC gene probe and the reference probe, centromer enumeration probe for chromosome 3(CEP3), were directly labeled with Spectrum Red_ and Spectrum Green_ fluorophore-conjugated dUTP provided by GP medical Corporation, China. FISH was performed on slides fixed with Carnoy fixing solution. After overnight hybridization at 42°C, slides were washed three times in 50% formamide/2× SSC at 46°C for 5minutes, followed by washed in 2× SSC (46°C, 10 minutes) and 0.1% NP40 in 2× SSC (46°C, 5 minutes). The slides were stained with nuclear stain (DAPI) and evaluated under a Zeiss epifluorescence microscope (Zeiss, Oberkochen, Germany Fluorescence Microscope Company) equipped with the corresponding wavelength filter, CCD camera, and image capturing and analyzing system. Signal copy numbers of both hTERC and 3q marker were counted from at least 200 nonoverlapping nuclei cells per case by two trained technologists blindly. Cases were considered positive for the hTERC assay when more than 5.8%, which amounts to mean value plus 3 fold of standard deviation of percentage of hTERC amplification cells in NNL group, of the cells exhibited a hTERC signal number more than 2. hTERC signals with the same number of CEP3 marker signals per nucleus in more than 80% of cells were considered normal [17].

### Immunohistochemistry for hTERT

Immunohistochemical staining for the hTERT expression was completed following standard IHC procedures. In brief, the 5-μm paraffin sections cut on poly-L-lysine-coated microscopy slides were first deparaffinized and rehydrated in graded alcohols. The sections were heated in citrate buffer (0.01 M, pH 6.0, DAKO Target Retrieval Solution) in a microwave oven (95°C, 18 min), followed by blocking the non-specific binding sites with normal rabbit serum. Sections were incubated with the primary antibody, antihuman hTERT antibody (PC563) (EMD Biosciences, Merck KGaA, Darmstadt, Germany) in a humidified chamber over night at 4°C (dilution 1:40). This purified rabbit polyclonal (IgG) antibody has been raised against the synthetic peptide corresponding to amino acids 348–358 of human hTERT, and recognizes a 128 kDa human hTERT protein. Slides were then processed with universal LSAB2-2 single reagents (peroxidase) kit (Dako), and expression of hTERT was localized by incubation with DAB (diaminobenzidine). As a final step, the slides were stained with a light hematoxylin counterstaining. Negative controls were similarly processed by omitting the primary antibody, and biopsies from breast cancer were used as positive controls.

### TRAP-PCR for quantification of telomerase activity

Biopsy samples were stored at −70°C. The extraction of telomerase protein and evaluation of its activity were measured by the telomeric repeat amplification protocol (TRAP) method using the TRAPeze_ XL telomerase detection kit (Chemicon International, Inc., Temecula, CA), as described previously [13]. Briefly, extracts from 40 to 100 mg of frozen cervical tissues were homogenized in approximately 100–200 μl of CHAPS lysis buffer. After 30 min of incubation on ice, the suspensions were centrifuged at 12,000g for 20 min at 4°C, after which the supernatant was frozen rapidly and stored at −70°C. Aliquot of extract containing 1μg of protein was used for each assay. The PCR condition was as follows: After 30 min for telomerase extension in 30°C, 95°C for 5 min to activate the Taq polymerase; 36 cycles of 94°C for 30 s, 55°C for 30 s, and 72°C for 60 s; an extension at 72°C for 3 min and then at 55°C for 25 min; finally a 4°C incubation. A fluorescent plate reader (Wallac 1420 ARVOsx, Perkin-Elmer,Wellesley, MA) was used to detect the levels of fluorescein and sulphorhodamine. The level of telomerase activity was expressed as total product generated (TPG) units. All samples were run in duplicate. A reaction mixture with 2μl of CHAPS lysis buffer instead of sample extract was used as the negative control.

### Statistical analysis

Statistical analyses were performed using the SPSS software packages (SPSS for Windows, version 15.0). Fisher’s exact test was used to compare the frequency of hTERC gene–amplified samples in the defined biopsy groups. Chi-square test, one-way analysis of variance (ANOVA) and logistic correlation analysis were used when appropriate. In all tests, values of P < 0.05 were regarded statistically significant.

## Results

### Genomic amplification of hTERC

**Figure 1:**
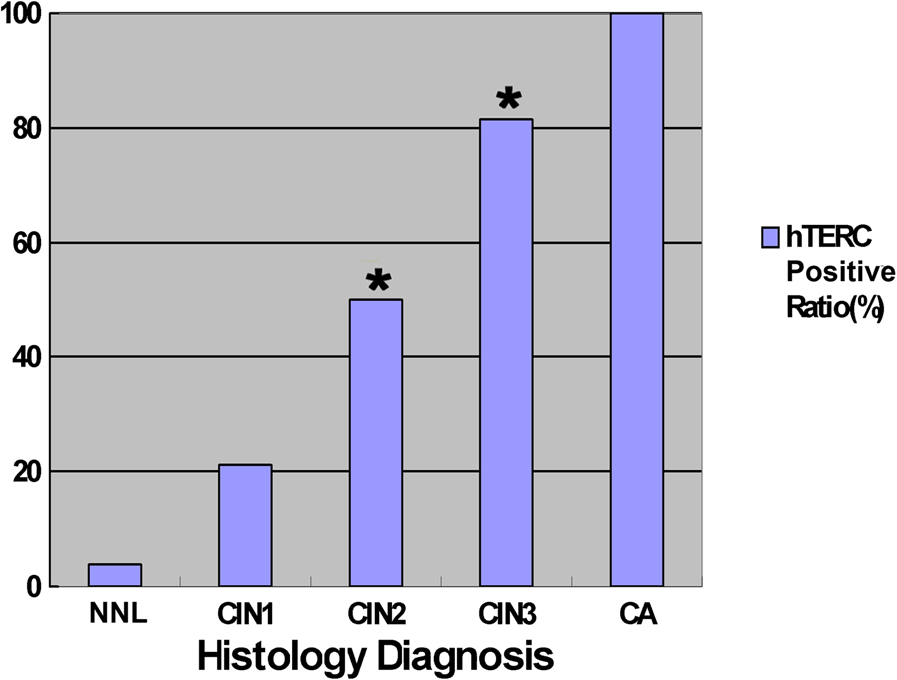
**Graph depicting the distribution of hTERC amplification by histological diagnosis.** *The group of CIN2 and CIN3 samples revealed a statically significant increase with hTERC gain per slide (p=0.022, p=0.009, respectively).

**Figure 2:**
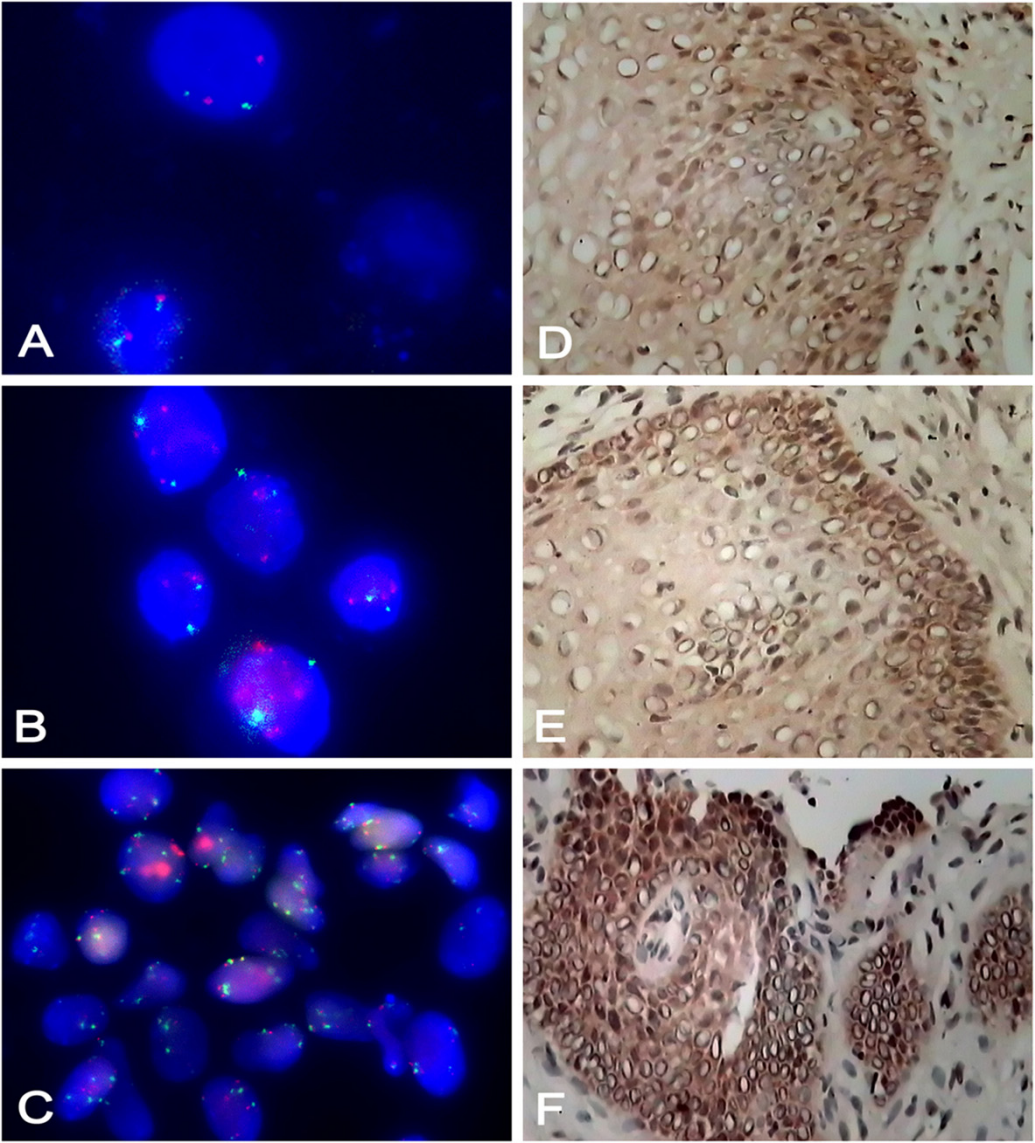
**Double-color probe set for the detection of the hTERC gain and immunohistological detection for hTERT in cervical dysplasia.A**. interphase FISH shows a normal signal pattern containing two copies each of the hTERC ( red) and CEP3 (green) (X630). **B**. interphase FISH shows increased number of signals for hTERC (red) compared to CEP3(green) (X630) **C**. interphase FISH shows gain of both hTERC (red) and CEP3 (green) gains in the larger cells (X630). **D**. Weakly hTERT positive cells are found in the suprabasal cell layers. (IHC for hTERT, original magnification× 400) E. A high-grade CIN3 lesion, where hTERT positive cells are found throughout the full thickness of the epithelium. (IHC for hTERT, original magnification×400) **F**. An invasive squamous cell carcinoma with strong hTERT expression. The staining intensity varies from weak to intense dark brown, indicating that the expression levels of hTERT vary from cell to cell. (IHC for hTERT, original magnification× 400).

FISH analysis was performed on 114 cervical exfoliated specimens, and 11 of them were excluded because of the lack of hybridization signals or too few cells on slides. The percentage of successfully hybridized samples was 96.49%. The results of the enumerations were summarized in Table 1 and Figures 1, 2.-A, B, C shows representative hybridizations. This analysis reveals that the average number of hTERC-positive cases increases accordingly with escalating levels of dysplasia. The percentage of hTERC-positive cases found on slides from NNL and CIN1 categories was similar. The group of CIN2 and CIN3 samples, however, revealed a statically significant increase with hTERC gain per slide (p=0.022, 0.009, respectively).

**Table 1:** Summary of hTERC amplification according to histology diagnosis

**Histology Dx**	**Number of cases**	No. of cases with hTERC amplification (%)*	**Mean of cell number with hTERC amplification**
NNL	26	0	1.058
CIN1	19	4 (21.1%)	5.132
CIN2	12	6 (50%)	6.083
CIN3	27	22 (81.5%)	11.093
CA	19	19 (100%)	21.105

Dx, diagnosis; NNL, non neoplastic lesion; CIN, cervical intraepithial neoplasia; CA, cervical carcinoma.

*A case of hTERC amplification was defined as more than 5.8% of the cells showed a pattern when number of hTERC signal was more than 2.

**Figure 3:**
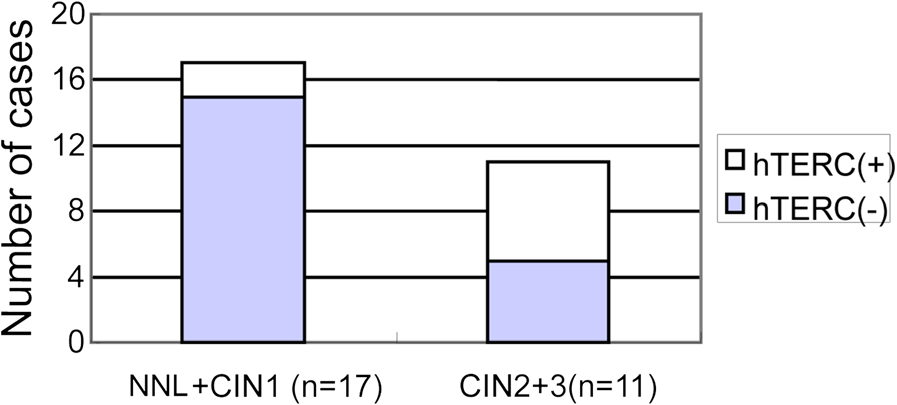
**Box-plot of number of cases with/without hTERC amplification by biopsy diagnosis for patients with a cytologic diagnosis of LSIL.** The number of hTERC amplification positive cases was differentiate between NNL+CIN1 group and CIN2 + CIN3 group. (P=0.03).

Statistical analysis showed that in 28 cases diagnosed as LSIL on cytology, the positive cases with hTERC amplification differentiated those histological diagnosed as high-grade dysplasia (CIN2/3) from those diagnosed as low-grade dysplasia (CIN1) or NNL (P=0.03) (Figure 3).

### hTERT protein expression

We found biopsies with normal epithelium were completely negative for hTERT, or at maximum, showing scattered positive cells within the parabasal layers. Somewhat more hTERT positive staining was observed in metaplastic squamous epithelium, but only in single cells. The staining pattern was completely different in CIN lesion; there was usually full thickness of the lesion demonstrated positive hTERT expression, varying in intensity from weak to extremely strong. Positive hTERT staining was mostly confined to nuclei only, but on several occasions, also weak cytoplasmic expression was detectable (Figure 2-D, E, F).

### Telomerase activity

**Figure 4:**
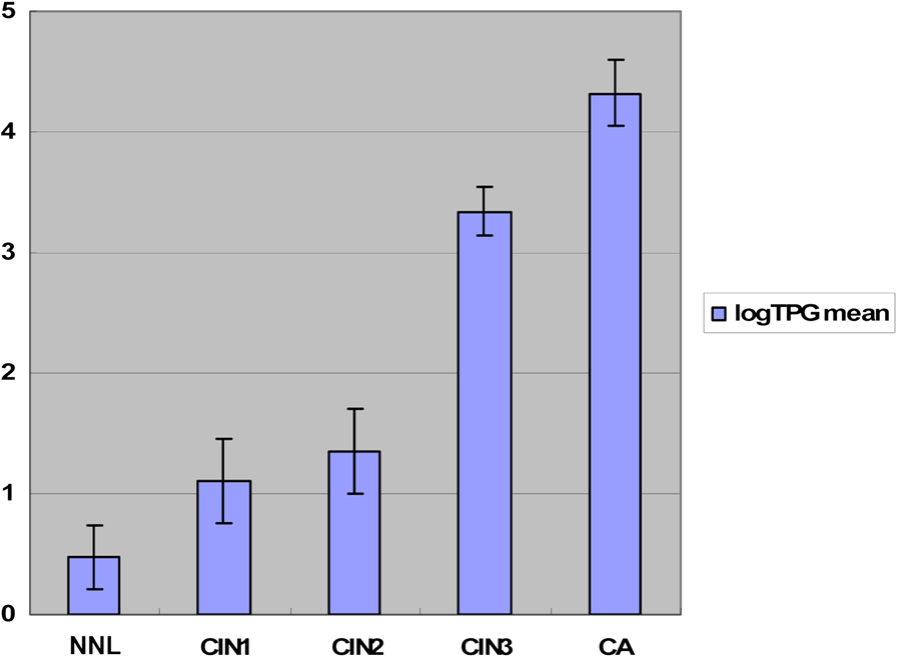
**Quantification of telomerase activity in different biopsy groups.** The vertical axis represents the log_10_ [TPG] levels for telomerase activity in the samples. Telomerase activity showed no difference neither in CIN1 to NNL groups (P=0.153), nor in CIN2 to CIN1 groups (p=0.619). CIN3 exhibited high levels of telomerase activity compared with CIN2 group (P=0.000). Samples with CA expressed higher levels of telomerase activity than with CIN3 (P=0.006).

The telomerase activity in different histological groups is summarized in Figure 4. We found that telomerase activity was higher in CIN3/CA compared to control normal or CIN1/2(P=0.00); The levels of telomerase activity were 0.473±0.259 in normal group, 1.104±0.347 in CIN1, 1.350±0.347 in CIN2, 3.342±0.194 in CIN3, 4.318±0.274 log_10_[TPG] in CA. Therefore, we speculate that variations in telomerase activity among individual histology groups could indicate to their severity of dysplasia.

We looked for an association of hTERC gain with the telomerase activity using logistic correlation analysis. The Pearson correlation is 0.594 (P=0.000).

### Association of the hTERC amplification with HPV Infection

**Figure 5:**
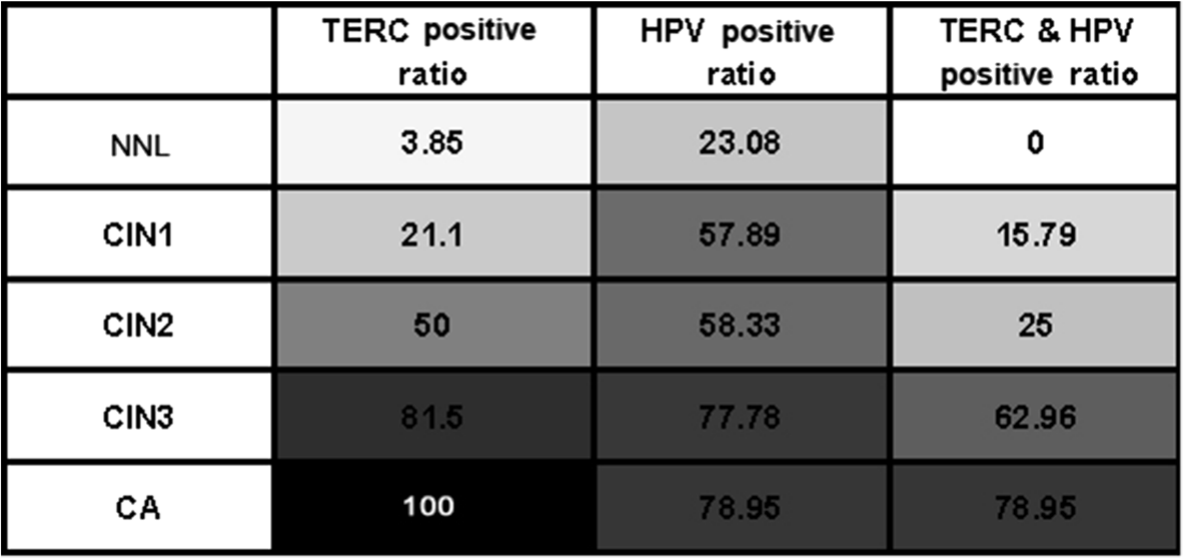
**Gray-scale graph labels the percentage of positive cases in individual groups.** The color turns from white to gray, and lastly to black, demonstrates the increasing percentage of positive cases.

114 cervical specimens included in the study had previously been analyzed for the presence of HPV DNA by HC2 test. 77 of them were found infected with high-risk HPV types. The results for HPV-positive, hTERC-amplification and both HPV and hTERC positive ratio in cervical specimens grouped by histology diagnosis were shown in Figure 5.

This analysis reveals that both HPV-infected positive ratio and hTERC-amplification positive ratio increase accordingly with escalating levels of dysplasia. hTERC positive ratio of patients classified as histologically NNL was just 3.85%, whereas those of classified CIN3 and CA were 81.5% and 100%, respectively. The difference of the hTERC positive ratio in CIN2 and CIN3 categories was statistically significant (p=0.009).

The HPV-infected status was also determined using HC2 for all of the different histological categories. The results revealed that 23.08% of high-risk HPV positive patients were found in NNL group, and 78.95% in CA group. There was no statistically difference to be observed in CIN2/3/CA groups (p=0.232, 0.297, respectively).

To further investigate the relationships between HPV infection and hTERC amplification, we analyzed the data combined. Not all genomic abnormal cases were found to be positive by HPV, and not all HPV-positive cases showed signs of hTERC amplification. Over all, the advancement of the disease correlated well with the increased genomic instability in HPV-infected cases. A marked increase in the accumulation of both HPV& hTERC positive cases was observed in the CIN3 subgroup compared with the CIN2 group, 62.96% versus 25%, respectively. The difference was statistically significant (p=0.007). Interestingly, the subgroup of patients with NNL diagnosis did not have any case to be shown as both HPV and hTERC positive.

## Discussion

Genomic amplification of the hTERC in cytologic specimens was significantly correlated with the severity of dysplasia. Patients with biopsy diagnoses of CIN or CA had significantly higher percentages of cells with extra copies of hTERC than did patients with a normal biopsy diagnosis. This test may be a complementary to regular screening methods to detect patients with high-grade lesions.

Several authors have shown that the percentage of cells with gain of hTERC copy number increased with the severity of dysplasia [5,6,9-12,18]. Heselmeyer et al. analyzed 57 thin-layer slides by FISH for the visualization of chromosomal copy number changes and found 63% of the HSIL( CIN2) lesions and 76% of the HSIL(CIN3) lesions showed extra copies of 3q [5]. In another paper, the same group designed a retrospective study set to 59 previously stained Pap smears, and proposed that gains of chromosome arm 3q play a crucial role in the progression of severe dysplasia/carcinoma in situ to invCA [6]. One study else on tissue sections also showed that 3q gain was detected in all of 12 cervical adenocarcinomas [8]. In our study, we analyzed a series of 114 Chinese women. Based on intensive test of exfoliated cell samples, our data show that extra copy numbers of hTERC present in 50% of CIN2, 81.5% of CIN3 and 100% of CA, no matter of what kind of histology type, of cases respectively. This result was similar to other studies in China [18,19]. The percentage of successfully hybridized samples was 96.49%, considerably higher than in the series of Pap smears [5].

Hongxiu Han et al. [20] suggested helpful biological markers would be in need of distinguishing CIN1 from CIN2/3. The expression of hTERT was evaluated as a potential marker of the high-grade premalignant CIN 2/3 lesions [14]. A recent study showed that overexpression of hTERC and hTERT together in vitro greatly induced telomerase activity and telomere length elongation, while stable overexpression of either hTERC or hTERT alone induced telomerase activity and telomere length elongation to a lesser extent [21]. Consistent with this study, several studies also highlighted the fact that both telomerase components restrict telomerase activity and telomere length in vitro, suggesting that the tumorigenic effects of hTERT overexpression are reliant on hTERC expression and in fact hTERT overexpression in an hTERC deficient background has anti-tumourigenic effects [22-24]. Our data showed that in cervical intraepithelial lesions, levels of hTERC amplification were correlated with telomerase activity and hTERT expression. Furthermore, comparing with biopsy procedure, exfoliated cells samples used for hTERC testing in our study were easier to get from patients with a concurrent physical examination. From this point of view, we suggest there is the potential value of detecting hTERC gain by FISH method for distinguishing between lesions with and without progression to in situ or invasive malignancy of uterine cervix.

In a previous study by Sokolova I et al. [11], 235 liquid-based preparation from cervical specimens were analyzed for aberrations of 3q26 using a commercially two-color FISH probe. They found in cases diagnosed as LSIL on cytology, the percentage of cells with gain of 3q26 did not differentiate those histologically diagnosed as high-grade dysplasia (CIN2/3) from those diagnosed as low-grade dysplasia (CIN1). Unfortunately, only a few cases had both HPV and FISH testing from one vial in that study. As shown in our study, we not only analyzed abnormal cells on exfoliated cervicovaginal preparations for gain of hTERC using a FISH probe similar to that used by Nancy P et al. [12], but also each case had both HPV testing and concurrent biopsy results. In cases of LSIL, gain of 3q26 differentiated those lesions that had CIN2/3 on biopsy from those that did not.

It is not sufficient for HPV screening assays by itself in predicting the progression from atypical or mild dysplasia to high-grade lesion and subsequently to cancer, because this screening is lack of a high positive predictive value for the presence of dysplasia. Furthermore, a report indicated that some kind of cervical cancer, such as minimal deviation adenocarcinoma, was not associated with high-risk HPV [25]. Qisang et al. [26] reported a significant positive correlation between positive hTERC gain and HR-HPV infection, histopathologic lesions (all P<0.01) in patients with cervical disease. Our study demonstrates that there are 23% HPV-infected positive cases in NNL, and 78.95% in CA group. From the gray-scale graph, we can see the HPV position ratio increase according to the severity of dysplasia. However, the predictive value of hTERC amplification test is much better than HPV test. Only 3.85% hTERC amplification positive ratio was observed in NNL group, and 100% in CA group, including two cases of minimal deviation adenocarcinoma. If combined these two tests together, it seems like much better than any one of them alone. In NNL group, there was not any case with both HPV and hTERC positive. From the clinical perspective, we hypothesize that hTERC gain, combining with testing of HPV infection by HC2, might have a very significant clinical application.

In summary, this study presents gain of hTERC in exfoliated cervicovaginal epithelial specimen and suggests a role of the factor in the biology of cervical cancer. Potential clinical usefulness of the hTERC FISH would be to distinguish patients with clinically significant cervical lesions from those that are insignificant lesions with a high risk of progression from those with a low risk of progression or an adjunct to cytology screening, especially in HPV-infection patients. However, more studies are needed to determine the application of hTERC gain in surveillance and prognosis of invasive cervical cancer.

## Competing interests

The authors declare that they have no conflicts of interest to disclose.

## Authors’ contributions

HL: study design, experimental studies, data analysis and manuscript preparation. SL: study design and manuscript review. HW: the guarantor of integrity of the entire study, study design, experimental studies, data analysis and manuscript preparation. XX:clinical studies, data analysis and manuscript preparation. XC: experimental studies and dataanalysis. XZ: experimental studies and manuscript review. YZ: clinical studies and manuscript review. All authors read and approved the final manuscript.

## References

[B1] What are the key statistics about Cervical Cancer? http://www.cancer.org/Cancer/CervicalCancer/DetailedGuide/cervical-cancer-key-statistics.

[B2] Song M Lin AN Study on the mortality trend of female cervical cancer from 1996 to 2005 in China Xiandai Yufang Yixue 2009 36 47 50

[B3] Ostor AG Mulvany N The Pathology of Cervical Neoplasia Curr Opin Obstet Gynecol 1996 8 69 73 8777262

[B4] Cohen SB Graham ME Lovrecz GO Bache N Robinson PJ Reddel RR Protein composition of catalytically active human telomerase from immortal cells Science 2007 315 1850 1853 10.1126/science.1138596 17395830

[B5] Heselmeyer-Haddad K Janz V Castle PE Chaudhri N White N Wilber K Morrison LE Auer G Burroughs FH Sherman ME Ried T Detection of genomic amplification of the human telomerase gene (TERC) in cytologic specimens as a genetic test for the diagnosis of cervical dysplasia Am J Pathol 2003 163 1405 1416 10.1016/S0002-9440(10)63498-0 14507648 PMC1868295

[B6] Heselmeyer-Haddad K Sommerfeld K White NM Chaudhri N Morrison LE Palanisamy N Wang ZY Auer G Steinberg W Ried T Genomic Amplification of the Human Telomerase Gene (TERC) in Pap Smears Predicts the Development of Cervical Cancer Am J Pathol 2005 166 1229 1238 10.1016/S0002-9440(10)62341-3 15793301 PMC1602397

[B7] Pao CC Tseng CJ Lin CY Yang FP Hor JJ Yao DS Hsueh S Differential expression of telomerase activity in human cervical cancer and cervical intraepithelial neoplasia lesions J Clin Oncol 1997 15 1932 1937 9164204 10.1200/JCO.1997.15.5.1932

[B8] Anderson S Shera K Ihle J Billman L Goff B Greer B Tamimi H McDougall J Klingelhutz A Telomerase activation in cervical cancer Am J Pathol 1997 151 25 31 9212727 PMC1857907

[B9] Andersson S Wallin KL Hellstrom AC Morrison LE Hjerpe A Auer G Ried T Larsson C Heselmeyer-Haddad K Frequent gain of the human telomerase gene TERC at 3q26 in cervical adenocarcinomas Br J Cancer 2006 95 331 338 10.1038/sj.bjc.6603253 16847471 PMC2360637

[B10] Hopman A Theelen W Hommelberg P Kamps M Herrington CS Morrison LE Speel EJ Smedts F Ramaekers FC Genomic integration of oncogenic HPV and gain of the human telomerase gene TERC at 3q26 are strongly associated events in the progression of uterine cervical dysplasia to invasive cancer J Pathol 2006 210 412 419 10.1002/path.2070 17054308

[B11] Sokolova I Alicia A-S Song MH Sitailo S Policht F Kipp BR Voss JS Halling KC Ruth A King W Underwood D Brainard J Morrison L Chromosomal biomarkers for detection of human papillomavirus associated genomic instability in epithelial cells of cervical cytology specimens J Mol Diagn 2007 9 5 604 611 10.2353/jmoldx.2007.070007 17975027 PMC2049051

[B12] Nancy PC Khanna A Marilyn D Guo M Guo N Lin E Katz RL Gain of the 3q26 region in cervicovaginal liquid-based pap preparations is associated with squamous intraepithelial lesions and squamous cell carcinoma Gynecol Oncol 2008 110 37 42 10.1016/j.ygyno.2008.01.040 18433848

[B13] Hashimoto Y Murakami Y Uemura K Hayashidani Y Sudo T Ohge H Fukuda E Sueda T Hiyama E Detection of human telomerase reverse transcriptase (hTERT) expression in tissue and pancreatic juice from pancreatic cancer Surgery 2008 143 113 125 10.1016/j.surg.2007.07.042 18154939

[B14] Saha B Chaiwun B Tsao-Wei DD Groshen SL Naritoku WY Atkinson RD Taylor CR Imam SA Telomerase and markers of cellular proliferation are associated with the progression of cervical intraepithelial neoplasia lesions Int J Gynecol Pathol 2007 26 214 222 10.1097/01.pgp.0000250146.44592.d2 17581401

[B15] Brance M Giorgi C Ciotti M Santini D Bonito LD Costa S Benedetto A Bonifacio D Di Bonito P Paba P Accardi L Mariani L Ruutu M Syrjanen S Favalli C Syrjanen K Upregulation of telomerase (hTERT) is related to the grade of cervical intraepithelial neoplasia, but is not an independent predictor of high-risk human papillomavirus, virus persistence, or disease outcome in cervical cancer Diagn Cytopathol 2006 34 739 748 10.1002/dc.20554 17041957

[B16] Thiberville C Guillaud M Lockwood W Lam W Follen M MacAulay C Multi-scale system biology applied to cervical inter-epithelial neoplasia Gynecol Oncol 2007 107 Suppl 1 S72 S82 17868784 10.1016/j.ygyno.2007.07.047

[B17] Zhang A Maner S Betz R Angstrom T Stendahl U Bergman F Zetterberg A Wallin KL Genetic alterations in cervical carcinomas: frequent low-level amplifications of oncogenes are associated with human papillomavirus infection Int J Cancer 2002 101 427 433 10.1002/ijc.10627 12216070

[B18] Chen S Yang Z Zhang Y Qiao Y Cui B Zhang Y Kong B Genomic amplification patterns of human telomerase RNA gene and C-MYC in liquid-based cytological specimens used for the detection of high-grade cervical intraepithelial neoplasia Diagn Pathol 2012 7 40 10.1186/1746-1596-7-40 22500694 PMC3379933

[B19] Jin Y Li JP He D Tang LY Zee CS Guo SZ Zhou J Chen JN Shao CK Clinical Significance of Human Telomerase RNA Gene (hTERC) Amplification in Cervical Squamous Cell Lesions Detected by Fluorescence in Situ Hybridization Asian Pacific J Cancer Prev 2011 12 1167 1171 21875260

[B20] Han H Yang Y Lu Z He Q Lin Z Decreased D2-40 and increased p16INK4A immunoreactivities correlate with higher grade of cervical intraepithelial neoplasia Diagn Pathol 2011 6 59 10.1186/1746-1596-6-59 21729305 PMC3157410

[B21] Cristofari G Lingner J Telomere length homeostasis requires that telomerase levels are limiting EMBO J 2006 25 565 574 10.1038/sj.emboj.7600952 16424902 PMC1383536

[B22] Gonzalez-Suarez E Samper E Ramirez A Flores JM Martin-Caballero J Jorcano JL Blasco MA Increased epidermal tumors and increased skin wound healing in transgenic mice overexpressing the catalytic subunit of telomerase, mTERT, in basal keratinocytes EMBO J 2001 20 2619 2630 10.1093/emboj/20.11.2619 11387197 PMC125492

[B23] Gonzalez-Suarez E Samper E Flores JM Blasco MA Telomerase deficient mice with short telomeres are resistant to skin tumorigenesis Nat Genet 2000 26 114 117 10.1038/79089 10973262

[B24] Cayuela L Flores JM Blasco MA The telomerase RNA component Terc is required for the tumour-promoting effects of Tert overexpression EMBO Rep 2005 6 268 274 10.1038/sj.embor.7400359 15731767 PMC1299269

[B25] Gong L Zhang W-D Liu X-Y Han X-J Yao L Zhu S-J Lan M Li Y-H Zhang W Clonal status and clinicopathological observation of cervical minimal deviation adenocarcinoma Diagn Pathol 2010 5 25 10.1186/1746-1596-5-25 20416098 PMC2877003

[B26] Qisang G Long S Youji F Cervical cancer screening: hTERC gene amplification detection by FISH in comparison with conventional methods Open J Obstet Gynecol 2012 2 11 17 10.4236/ojog.2012.21003

